# Trauma complications and in-hospital mortality: failure-to-rescue

**DOI:** 10.1186/s13054-020-02951-1

**Published:** 2020-05-15

**Authors:** Toshikazu Abe, Akira Komori, Atsushi Shiraishi, Takehiro Sugiyama, Hiroki Iriyama, Takako Kainoh, Daizoh Saitoh

**Affiliations:** 1 Department of Health Services Research, Faculty of Medicine, University of Tsukuba, 1-1-1 Tennodai, Tsukuba, Ibaraki 305-8577 Japan; 2 Department of Emergency and Critical Care Medicine, Tsukuba Memorial Hospital, Tsukuba, Japan; 3 Department of General Medicine, Juntendo University, Tokyo, Japan; 4 Emergency and Trauma Center, Kameda Medical Center, Kamogawa, Japan; 5 Diabetes and Metabolism Information Center, Research Institute, National Center for Global Health and Medicine, Tokyo, Japan; 6 Department of Public Health/Health Policy, Graduate School of Medicine, The University of Tokyo, Tokyo, Japan; 7 Department of Traumatology and Emergency Medicine, National Defense Medical College, Tokorozawa, Japan

**Keywords:** Injuries, Hospital mortality, Indicators, Quality

## Abstract

**Background:**

Reducing medical errors and minimizing complications have become the focus of quality improvement in medicine. Failure-to-rescue (FTR) is defined as death after a surgical complication, which is an institution-level surgical safety and quality metric that is an important variable affecting mortality rates in hospitals. This study aims to examine whether complication and FTR are different across low- and high-mortality hospitals for trauma care.

**Methods:**

This was a retrospective cohort study performed at trauma care hospitals registered at Japan Trauma Data Bank (JTDB) from 2004 to 2017. Trauma patients aged ≥ 15 years with injury severity score (ISS) of ≥ 3 and those who survived for > 48 h after hospital admission were included. The hospitals in JTDB were categorized into three groups by standardized mortality rate. We compared trauma complications, FTR, and in-hospital mortality by a standardized mortality rate (divided by the institute-level quartile).

**Results:**

Among 184,214 patients that were enrolled, the rate of any complication was 12.7%. The overall mortality rate was 3.7%, and the mortality rate among trauma patients without complications was only 2.8% (non-precedented deaths). However, the mortality rate among trauma patients with any complications was 10.2% (FTR). Hospitals were categorized into high- (40 facilities with 44,773 patients), average- (72 facilities with 102,368 patients), and low- (39 facilities with 37,073 patients) mortality hospitals, using the hospital ranking of a standardized mortality rate. High-mortality hospitals showed lower ISS than low-mortality hospitals [10 (IQR, 9–18) vs. 11 (IQR, 9–20), *P* < 0.01]. Patients in high-mortality hospitals showed more complications (14.2% vs. 11.2%, *P* < 0.01), in-hospital mortality (5.1% vs. 2.5%, *P* < 0.01), FTR (13.6% vs. 7.4%, *P* < 0.01), and non-precedented deaths (3.6% vs. 1.9%, *P* < 0.01) than those in low-mortality hospitals.

**Conclusions:**

Unlike reports of elective surgery, complication rates and FTR are associated with in-hospital mortality rates at the center level in trauma care.

## Key points

- In this retrospective cohort study, patients in low-performance hospitals showed more complications, in-hospital mortality, FTR, and non-precedented deaths than those in high-performance hospitals, unlike reports of elective surgery.
- A lower risk of complications and better care of those with complications could play crucial roles in trauma care.

## Background

Reducing medical errors and minimizing complications have become the focus of quality improvement in the medical field [1]. Failure-to-rescue (FTR) is defined as death after a surgical complication [2]. Regarding elective surgery, a study showed that at the hospital level, complications and mortality were not correlated, but FTR and mortality were correlated [3]. Therefore, the focus should not be on improvising operative techniques to prevent complications but on more efficient rescuing from the complications. Thus, FTR is an institution-level surgical safety and quality metric [4] and is considered an important variable affecting mortality rates in hospitals [5]; this metric indicates the ability of a hospital to identify and successfully manage complications [6].

Recently also in a case of trauma, FTR was found to be an important variable [7] because it is more about an institution’s ability to rescue those who develop complications [8–10]. Nevertheless, whether FTR in trauma care contributes to variations in mortality across centers [7] remains debatable, as there are some concerns regarding the use of FTR as a quality measure of trauma care [11]. First, severe trauma patients die in the hours immediately after injury, although all patients after elective surgery ideally survive. Outcomes after trauma complications may be less modifiable. FTR might play a relatively minor role in trauma patients compared with those after elective surgery. Moreover, with rapid progress in endovascular interventions and intensive care, surgeries for trauma have reduced. Conceivably, complications and FTR should be important in trauma patients regardless of FTR playing a minor or major role. Therefore, our aim was to investigate the association between a complication rate and FTR and a hospital performance level of trauma care in hospitals.

## Methods

### Design and setting

This was a retrospective cohort study using the Japan Trauma Data Bank (JTDB), which is a nationwide trauma registry established in 2003 by the Japanese Association for the Surgery of Trauma and by the Japanese Association for Acute Medicine with the aim of improving and ensuring the quality of trauma care in Japan, and compiled by the JTDB investigators [12]. A total of 264 hospitals, including 95% of the tertiary emergency medical centers in Japan, participated in the JTDB in 2017.

### Participants

Patients aged ≥ 15 years with an injury severity score (ISS) of ≥ 3 and diagnosed with trauma between 2004 and 2017 were enrolled in this study. Only patients who survived for > 48 h after hospital admission were included to exclude the impact of early deaths. Patients with pre-hospital or emergency department (ED) death, un-survivable [abbreviated injury scale (AIS) score of 6], burns, or unknown trauma mechanisms, missing data of in-hospital death, and hospital for > 2 years were excluded. Similar to a previous report [7], the current analysis was limited to hospitals contributing at least 200 patients to the cohort during the entire study period.

### Data collection

Data related to patient and hospital information in the JTDB include patient demographics, AIS, ISS, pre-hospital and in-hospital procedures, and clinical outcomes. Data collection was performed as part of the routine clinical patient management.

### Data definitions

**Fig. 1:**
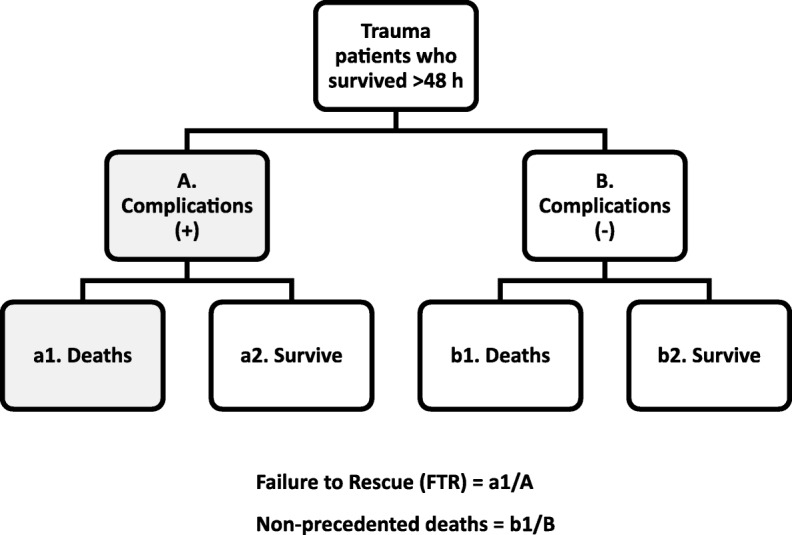
Conceptualization of the study. Among trauma patients who survived for > 48 h, some patients suffered from complications, while others did not; the proportion of patients with complications is the “complication rate,” which is one of the outcome indicators. Those who suffered from complications were more likely to die than those who did not suffer from complications, i.e., the rate of “failure-to-rescue (FTR),” which is another outcome indicator, is naturally more than the rate of “non-precedented deaths.” Overall in-hospital mortality comprises these components. However, unlike reports of elective surgery, it has been controversial whether complication rate or FTR explains more variations in in-hospital mortality. In addition, FTR in trauma complication studies includes not only patients who have undergone surgery but also those who have not undergone surgery; this is inconsistent with studies on elective surgery. We investigated this research question in the present study

The definition of complication was in accordance with the JTDB (Table 1), wherein FTR was defined as in-hospital mortality after at least one trauma complication. Non-precedented death was defined as patient death without any complications. Many trauma patients did not undergo surgical interventions, but FTR was considered in this study regardless of whether they underwent surgery, similar to a previous study [13]. Figure 1 shows the conceptualization of the study. The hospitals were separated into three groups by standardized mortality rate (hospital ranking).

**Table 1:** Complications of trauma patients according to the hospital ranking (hospital performance)

	Hospital outlier status			*P* value
	Low-mortality	Average-mortality	High-mortality	
Number of institutions	39	72	40	
Number of patients	37,073	102,368	44,773	
CNS				
Diabetes insipidus	89 (0.2)	226 (0.2)	139 (0.31)	< 0.01
Hydrencephalus	53 (0.1)	261 (0.3)	104 (0.2)	< 0.01
Fat embolism	22 (0.1)	189 (0.2)	32 (0.1)	< 0.01
Cerebrospinal fluid leakage	115 (0.3)	178 (1.2)	98 (0.2)	< 0.01
Meningitis	56 (0.2)	213 (0.2)	54 (0.1)	< 0.01
Higher brain dysfunction	765 (2.1)	2006 (2.0)	940 (2.1)	0.16
Mental disorders (PTSD et al.)	129 (0.4)	544 (0.5)	167 (0.4)	< 0.01
Others	315 (0.9)	1232 (1.2)	746 (1.7)	< 0.01
Circulation				
Acute coronary syndrome	10 (0.03)	78 (0.1)	35 (0.1)	< 0.01
Lethal arrhythmia	36 (0.1)	175 (0.2)	75 (0.2)	< 0.01
Acute kidney injury	86 (0.2)	286 (0.3)	160 (0.4)	< 0.01
Abdominal compartment syndrome	12 (0.03)	63 (0.1)	28 (0.1)	0.10
Others	251 (0.7)	580 (0.6)	327 (0.7)	< 0.01
Respiratory				
Lung edema	40 (0.1)	182 (0.2)	99 (0.2)	< 0.01
Atelectasis	466 (1.3)	1064 (1.0)	583 (1.3)	< 0.01
Pneumonia	990 (2.7)	3286 (3.2)	1572 (3.5)	< 0.01
Pulmonary embolism	88 (0.2)	640 (0.6)	72 (0.2)	< 0.01
Pyothorax	26 (0.1)	74 (0.1)	45 (0.1)	0.17
ARDS and respiratory failure	166 (0.5)	620 (0.6)	268 (0.6)	< 0.01
Others	137 (0.4)	354 (0.4)	206 (0.5)	< 0.01
Gastroenterology and hepato-biliary				
Ulcer and upper GI bleeding	87 (0.2)	573 (0.6)	157 (0.4)	< 0.01
Ileus	71 (0.2)	220 (0.2)	105 (0.2)	0.42
Pancreatitis	25 (0.1)	68 (0.1)	35 (0.1)	0.72
Cholecystitis	49 (0.1)	159 (0.2)	69 (0.2)	0.60
Hyperbilirubinemia and liver failure	48 (0.1)	175 (0.2)	79 (0.2)	0.18
Others	209 (0.6)	559 (0.6)	225 (0.5)	0.44
Bone and joint				
Compartment syndrome	63 (0.2)	411 (0.4)	126 (0.3)	< 0.01
Osteomyelitis	23 (0.1)	450 (0.4)	34 (0.1)	< 0.01
Refracture	17 (0.1)	376 (0.4)	14 (0.03)	< 0.01
Pseudoarthrosis	23 (0.1)	393 (0.4)	24 (0.1)	< 0.01
Others	75 (0.2)	336 (0.3)	160 (0.4)	< 0.01
Coagulation				
DIC and coagulation disorder	248 (0.7)	934 (0.9)	538 (1.2)	< 0.01
Thrombopenia (< 50,000)	93 (0.3)	383 (0.4)	284 (0.6)	< 0.01
Others	51 (0.1)	176 (0.2)	261 (0.6)	< 0.01
Infection et al.				
Bacteremia	120 (0.3)	379 (0.4)	192 (0.4)	0.05
Sepsis or MOF	152 (0.4)	745 (0.7)	332 (0.7)	< 0.01
Abdominal abscess	35 (0.1)	115 (0.1)	37 (0.1)	0.23
UTI	582 (1.6)	1532 (1.5)	601 (1.3)	0.02
Infectious colitis	26 (0.1)	101 (0.1)	62 (0.1)	< 0.01
Wound infection	333 (0.9)	1528 (1.5)	464 (1.0)	< 0.01
Wound disruption	89 (0.2)	323 (0.3)	102 (0.2)	< 0.01
Decubitus	158 (0.4)	411 (0.4)	282 (0.6)	< 0.01
Hypothermia (< 35 °C)	49 (0.1)	173 (0.2)	203 (0.5)	< 0.01
Drug allergy	41 (0.1)	117 (0.1)	51 (0.1)	0.98
Others	232 (0.6)	972 (1.0)	442 (1.0)	< 0.01
Any complications	4164/37,073 (11.2)	12,838/102,368 (12.5)	6346/44,773 (14.2)	< 0.01

*PTSD* post-traumatic stress disorder, *ARDS* acute respiratory distress syndrome, *GI* gastrointestinal, *DIC* disseminated intravascular coagulation, *MOF* multiple organ failure, *UTI* urinary tract infection

### Analysis

To investigate the association between a hospital ranking (hospital performance level of trauma care) and trauma complications, FTR, and in-hospital mortality, the hospitals were ranked low, average, or high by standardized mortality rates. First, we performed a logistic regression model to predict the probability of deaths [P_p_(E)] after adjusting for baseline patient and trauma characteristics, which included patient’s age, sex, mechanism of injury, ISS, and vital signs at ED (Glasgow Coma Scale, systolic blood pressure, and heart rate). These variables were chosen based on clinical relevance and a previous study [7]. Next, the predicted probability of death for each patient at each hospital was summed to obtain a predicted mortality rate for each hospital [P_c_(E)]. In addition, we also calculated an observed in-hospital mortality rate [P_c_(O)] at each hospital. To yield a standardized mortality rate at each hospital, the overall mortality rate was multiplied by observed to expected [P_c_(O)/P_c_(E)] mortality ratio. Finally, hospitals were divided into three by the quartile of standardized mortality rate.

We compared the baseline characteristics, treatments, complications, and outcomes by the hospital ranking. Categorical variables were expressed as counts and percentages with comparisons performed using the chi-square test. Continuous variables were expressed as medians and interquartile ranges (IQRs) using the Kruskal–Wallis test because our study variables were not normally distributed.

We calculated the correlation coefficient between complication rate and FTR and in-hospital mortality and showed the correlation using bubble plots in all hospitals. As a sensitivity analysis, this correlation was analyzed based on the data from hospitals contributing at least 20 patients with complications in the cohort to avoid reporting bias.

All *P* values were two-sided, and *P* < 0.05 was considered statistically significant. We performed statistical analyses using the Stata software, version 15.1 (StataCorp, TX, USA). Bubble plots were drawn using JMP version 14.0 (SAS Institute, Cary, NC).

## Results

**Fig. 2:**
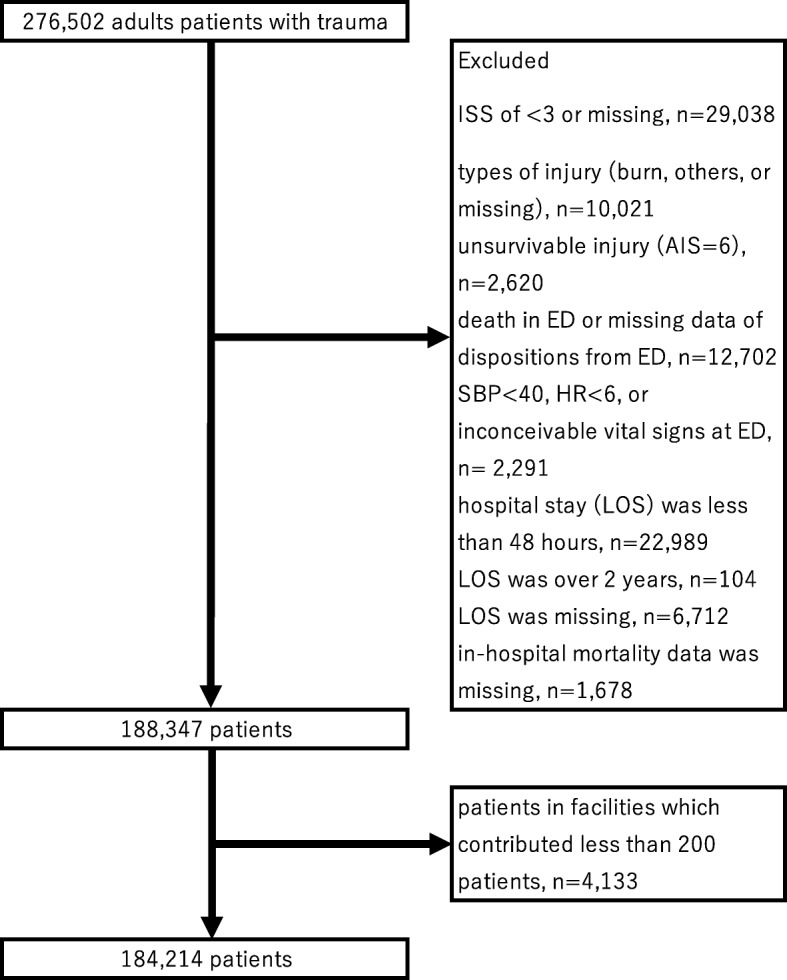
Participant selection

Of 294, 274 patients in the JTDB, there were 276, 502 adults (≥ 15 years) with trauma. Among these, 188, 347 met the inclusion and exclusion criteria, and after excluding patients in hospitals that contributed less than 200 patients to the JTDB, 184, 214 patients were analyzed in this study (Fig. 2).

The prevalence of any complication was 12.7%. The most frequent complications were pneumonia (3.2%), higher brain dysfunction (2.0%), urinary tract infection (1.5%), and atelectasis (1.2%). The overall mortality rate was 3.7%, and the mortality rate among trauma patients without complications was only 2.8% (non-precedented deaths). However, the mortality rate among trauma patients with any complications was 10.2% (FTR). Hospitals were categorized into high- (40 facilities with 44,773 patients), average- (72 facilities with 102,368 patients), and low- (39 facilities with 37,073 patients) mortality hospitals, using the hospital ranking (hospital performance).

Demographics and characteristics of trauma patients according to the hospital ranking (hospital performance) are shown in Table 2. High-mortality hospitals showed lower ISS than low-mortality hospitals [10 (IQR, 9–18) vs. 11 (IQR, 9–20), *P* < 0.01]. Treatments and interventions in trauma patients according to the hospital ranking (hospital performance) are shown in Table 3. Though patients in high-mortality hospitals received more emergency procedures in EDs than those in low-mortality hospitals (35.8% vs. 27.0%, *P* < 0.01), the former received fewer primary (50.4% vs. 54.7%, *P* < 0.01) and secondary surgeries (2.1% vs. 2.9%, *P* < 0.01) than the latter. Further, patients in high-mortality hospitals had more complications than those in low-mortality hospitals (14.2% vs. 11.2%, *P* < 0.01, Table 1).

**Table 2:** Demographics and characteristics of trauma patients according to the hospital ranking (hospital performance)

	Hospital outlier status			
	Low-mortality	Average-mortality	High-mortality	*P* value
Number of institutions	39	72	40	
Number of patients	37,073	102,368	44,773	
Age	65 (45–80)	65 (44–79)	64 (42–79)	< 0.01
Sex (male)	22,343 (60.3)	61,702 (60.3)	27,038 (60.4)	0.90
Type of injury				
Blunt (vs. penetrate)	35,983 (97.1)	99,391 (97.1)	43,340 (96.8)	< 0.01
AIS (≥ 3)				
Head	10,570 (28.5)	31,627 (30.9)	13,956 (31.2)	< 0.01
Face	389 (1.1)	758 (0.7)	250 (0.6)	< 0.01
Neck	178 (0.5)	398 (0.4)	149 (0.3)	< 0.01
Thorax	8268 (22.3)	22,635 (22.1)	9619 (21.5)	< 0.01
Abdomen and pelvis	2111 (5.7)	6067 (5.9)	2326 (5.2)	< 0.01
Spine	4286 (11.6)	11,048 (10.8)	4228 (9.4)	< 0.01
Upper extremity	2150 (5.8)	5917 (5.8)	1907 (4.3)	< 0.01
Lower extremity	11,300 (30.5)	34,763 (34.0)	15,289 (34.2)	< 0.01
Body surface	16 (0.04)	60 (0.1)	32 (0.1)	0.25
ISS	11 (9–20)	11 (9–19)	10 (9–18)	< 0.01
Vital signs at arrival				
GCS	15 (14–15)	15 (14–15)	15 (14–15)	< 0.01
SBP	137 (118–157)	138 (119–158)	139 (119–160)	< 0.01
HR	82 (71–95)	82 (72–95)	83 (72–96)	< 0.01
RR	20 (17–24)	20 (17–24)	20 (18–24)	< 0.01
BT	36.5 (36–37)	36.5 (36–37)	36.5 (36–37)	< 0.01
Alcohol	2803 (12.0)	9386 (14.2)	4638 (15.0)	< 0.01
Comorbidities				
Ischemic heart diseases	1620 (4.4)	4281 (4.2)	1702 (3.8)	< 0.01
Heart failure	1180 (3.2)	2322 (2.3)	966 (2.2)	< 0.01
Hypertension	9970 (26.9)	26,083 (25.5)	10,983 (24.5)	< 0.01
Other cardiac diseases	1582 (4.3)	4995 (4.9)	2143 (4.8)	< 0.01
Asthma	1044 (2.8)	3152 (3.1)	1388 (3.1)	< 0.01
COPD	243 (0.7)	746 (0.7)	310 (0.7)	0.329
Other chronic lung diseases	448 (1.2)	1018 (1.0)	505 (1.1)	< 0.01
Liver cirrhosis	267 (0.7)	728 (0.7)	366 (0.8)	0.08
Chronic hepatitis	596 (1.6)	1376 (1.3)	645 (1.4)	< 0.01
Peptic ulcer	530 (1.4)	2357 (2.3)	911 (2.0)	< 0.01
Inflammatory bowel diseases	253 (0.7)	676 (0.7)	170 (0.4)	< 0.01
Other gastrointestinal diseases	1568 (4.2)	3878 (3.8)	1637 (3.7)	< 0.01
DM	4152 (11.2)	11,207 (11.0)	4856 (10.9)	0.25
Obesity	47 (0.1)	108 (0.1)	71 (0.2)	0.03
Other metabolic diseases	1113 (3.0)	3342 (3.3)	1372 (3.1)	0.02
Stroke	2260 (6.1)	6243 (6.1)	2291 (5.1)	< 0.01
Psychiatric disease	1888 (5.1)	5600 (5.5)	3039 (6.8)	< 0.01
Dementia	2550 (6.9)	6771 (6.6)	2867 (6.4)	0.03
Other neurological diseases	834 (2.3)	2922 (2.9)	1287 (2.9)	< 0.01
HIV	8 (0.02)	22 (0.02)	6 (0.01)	0.57
Malignancies	875 (2.4)	2770 (2.7)	877 (2.0)	< 0.01
Hematological diseases	89 (0.2)	350 (0.3)	121 (0.3)	< 0.01
Chronic renal failure or HD	516 (1.4)	2452 (2.4)	507 (1.1)	< 0.01
Pregnancy	14 (0.04)	41 (0.04)	20 (0.04)	0.88
Others	1384 (3.7)	4750 (4.6)	2404 (5.4)	< 0.01
Steroid use	650 (1.8)	1629 (1.6)	658 (1.5)	< 0.01
Immunosuppressant use	170 (0.5)	358 (0.4)	119 (0.3)	< 0.01
Anticoagulant use	73 (0.2)	135 (0.1)	36 (01)	< 0.01
Previous healthy (no comorbidities reported)	16,893 (45.6)	45,356 (44.3)	20,037 (44.8)	< 0.01

Missing data: gender = 45, GCS = 14,509, SBP = 2705, HR = 6523, RR = 25,223, BT = 19,734, alcohol = 63,683

*AIS* abbreviated injury scale, *ISS* injury severity score, *GCS* Glasgow coma scale, *SBP* systolic blood pressure, *HR* heart rate, *RR* respiratory rate, *BT* body temperature, *COPD* chronic obstructive pulmonary disease, *DM* diabetes mellitus, *HIV* human immunodeficiency virus; hemodialysis

**Table 3:** Treatments and interventions of trauma patients according to the hospital ranking (hospital performance)

	Hospital outlier status			
	Low-mortality	Average-mortality	High-mortality	*P* value
Number of institutions	39	72	40	
Number of patients	37,073	102,368	44,773	
Blood transfusion within 24 h	5613 (15.4)	13,472 (13.5)	6276 (14.3)	< 0.01
Emergency procedures				
Oral intubation	2982 (8.0)	9246 (9.0)	5589 (12.5)	< 0.01
Nasal intubation	42 (0.1)	184 (0.2)	114 (0.3)	< 0.01
Cricothyroidotomy	44 (0.1)	114 (0.1)	60 (0.1)	0.51
Ventilator use	2925 (7.9)	6904 (6.7)	4562 (10.2)	< 0.01
Closed CPR	44 (0.1)	165 (0.2)	80 (0.2)	0.09
Open CPR	14 (0.04)	55 (0.1)	5 (0.01)	< 0.01
Aortic cross clamping	14 (0.04)	46 (0.04)	14 (0.03)	0.47
REBOA	73 (0.2)	295 (0.3)	137 (0.3)	< 0.01
Thoracentesis	63 (0.2)	181 (0.2)	128 (0.3)	< 0.01
Chest drainage	2012 (5.4)	6187 (6.0)	2867 (6.4)	< 0.01
Pericardial puncture	18 (0.1)	47 (0.1)	24 (0.1)	0.83
Pericardial fenestration	12 (0.03)	32 (0.03)	12 (0.03)	0.88
Shock pants use	8 (0.02)	20 (0.02)	5 (0.01)	0.46
Tourniquet use	40 (0.1)	171 (0.17)	99 (0.22)	< 0.01
Emergency craterization	240 (0.7)	678 (0.7)	372 (0.8)	< 0.01
Emergency TAE	868 (2.3)	3407 (3.3)	1270 (2.8)	< 0.01
Central venous line use	1247 (3.4)	3235 (3.2)	2141 (4.8)	< 0.01
Blood transfusion within 24 h	2754 (7.4)	8176 (8.0)	3608 (8.1)	< 0.01
Vasopressor use	454 (1.2)	1321 (1.3)	819 (1.8)	< 0.01
Open spine traction	76 (0.2)	177 (0.2)	117 (0.3)	< 0.01
Open bone traction	1834 (5.0)	5571 (5.4)	4650 (10.4)	< 0.01
External skeletal fixation	864 (2.3)	3043 (3.0)	1390 (3.1)	< 0.01
Other emergency bone fixation	1911 (5.2)	4389 (4.3)	1625 (3.6)	< 0.01
Primary surgeries				
Craniotomy	1410 (3.8)	3785 (3.7)	1751 (3.9)	0.14
Craterization	708 (1.9)	1505 (1.5)	610 (1.4)	< 0.01
Thoracotomy	211 (0.6)	655 (0.6)	289 (0.7)	0.281
Celiotomy	1156 (3.1)	2920 (2.9)	1229 (2.8)	< 0.01
Bone reduction and fixation	14,059 (37.9)	36,124 (35.3)	15,418 (34.5)	< 0.01
Revascularization	203 (0.6)	471 (0.5)	215 (0.5)	0.12
TAE	1169 (3.2)	3170 (3.1)	1423 (3.2)	0.69
Endoscopic surgery	85 (0.2)	157 (0.2)	78 (0.2)	0.01
Replantation of limbs and digits	131 (0.4)	298 (0.3)	183 (0.4)	< 0.01
Hemostasis	598 (1.6)	1221 (1.2)	609 (1.4)	< 0.01
Others	1925 (5.2)	3973 (3.9)	2073 (4.6)	< 0.01
Secondary surgeries				
Craniotomy	225 (0.6)	490 (0.5)	205 (0.5)	< 0.01
Craterization	55 (0.2)	119 (0.1)	41 (0.1)	0.06
Thoracotomy	40 (0.1)	79 (0.1)	25 (0.1)	0.03
Celiotomy	193 (0.5)	406 (0.4)	159 (0.4)	< 0.01
Bone reduction and fixation	497 (1.4)	1142 (1.1)	431 (1.0)	< 0.01
Revascularization	10 (0.03)	31 (0.03)	17 (0.04)	0.66
TAE	104 (0.3)	219 (0.22)	87 (0.2)	0.02
Endoscopic surgery	7 (0.02)	7 (0.01)	6 (0.01)	0.14
Hemostasis	31 (0.1)	98 (0.1)	53 (0.1)	0.28
Any interventions	23,770 (64.1)	62,326 (60.9)	28,083 (62.7)	< 0.01
Any emergency procedures	9993 (27.0)	30,011 (29.3)	16,037 (35.8)	< 0.01
Any primary surgeries	20,281 (54.7)	51,652 (50.0)	22,553 (50.4)	< 0.01
Any secondary surgeries	1079 (2.9)	2420 (2.4)	952 (2.1)	< 0.01

Emergency procedures: procedures performed during the emergency department stayed. Primary surgeries: surgeries performed at the first time. Any interventions = any primary surgeries or any secondary surgeries or any emergency procedures. Missing data: blood transfusion = 3891, primary surgeries (craniotomy = 158, craterization = 157, thoracotomy = 159, celiotomy = 159, bone reduction and fixation = 150, revascularization = 157, TAE = 157, endoscopic surgery = 159, replantation of limbs and digits = 159, hemostasis = 158, others = 155), secondary surgeries (craniotomy = 2011, craterization = 2011, thoracotomy = 2011, celiotomy = 2010, bone reduction and fixation = 2009, revascularization = 2011, TAE = 2011, endoscopic surgery = 2011, hemostasis = 2011)

*CPR* cardiopulmonary resuscitation, *REBOA* resuscitative endovascular balloon occlusion of the aorta, *TAE* transcatheter arterial embolization

**Fig. 3:**
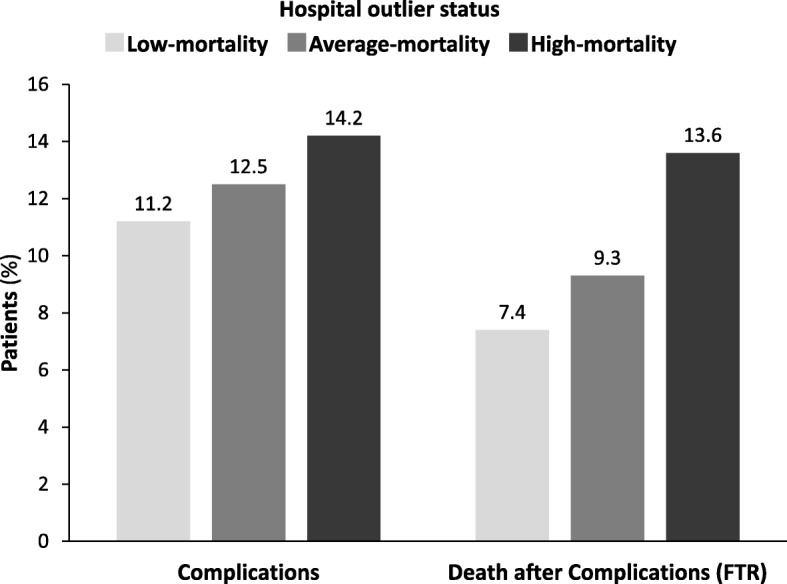
Complication rates and death after complications (failure-to-rescue) according to the hospital ranking (hospital performance). Complication rates and death after complications (failure-to-rescue) varied across the hospital ranking. The rate of death in patients with complications was almost twice as high in in-hospital patients with high mortality as in those with low mortality (7.4% vs. 13.6, *P* < 0.01)

Individual complications did not show a consistent pattern across the hospital ranking (hospital performance). Clinically, pneumonia, disseminated intravascular coagulation, coagulation disorder, and thrombocytopenia occurred more often in high-mortality hospitals than in low-mortality hospitals. An examination of outcomes according to the hospital ranking (hospital performance) (Table 4) revealed that high-mortality hospitals had significantly lower expected mortality than low-mortality hospitals did (3.7 ± 8.0% vs. 3.9 ± 8.5%, *P* < 0.01). However, in-hospital mortality (5.1% vs. 2.5%, *P* < 0.01), FTR (13.6% vs. 7.4%, *P* < 0.01), and non-precedented deaths (3.6% vs. 1.9%, *P* < 0.01) were higher in high-mortality hospitals than in low-mortality hospitals (Fig. 3).

**Table 4:** Outcomes of trauma patients according to the hospital ranking (hospital performance)

	Hospital outlier status			
	Low-mortality	Average-mortality	High-mortality	*P* value
Number of institutions	39	72	40	
Number of patients	37,073	102,368	44,773	
Expected mortality, mean ± SD	3.9 ± 8.5	3.7 ± 8.1	3.7 ± 8.0	< 0.01
In-hospital mortality, *n*/total (%)	942/37,073 (2.5)	3622/102,368 (3.5)	2264/44,773 (5.1)	< 0.01
Failure-to-rescue (FTR), *n*/total (%)				
(*n* = 23,348)	309/4164 (7.4)	1198/12,838 (9.3)	864/6346 (13.6)	< 0.01
Non-precedented deaths, *n*/total (%)				
(*n* = 160,866)	633/32,909 (1.9)	2424/89,530 (2.7)	1400/38,427 (3.6)	< 0.01
Admission, *n*/total (%)				
ICU	22,242 (60.0)	58,938 (57.6)	26,064 (58.2)	< 0.01
Ward	14,750 (39.8)	41,347 (40.4)	18,173 (40.6)	
Others	81 (0.2)	2083 (2.0)	536 (1.2)	
Place after discharge, *n*/total (%)				
Home	13,779 (37.2)	48,428 (47.4)	21,195 (47.4)	< 0.01
Another facility	21,687 (58.5)	48,095 (47.1)	20,398 (45.6)	
Others	650 (1.8)	2009 (2.0)	849 (1.9)	
Length of hospital stay, median (IQR), days	18 (10–31)	21 (10–37)	22 (11–39)	< 0.01

Missing: place after discharge = 269. Data was individual level

*SD* standard deviation, *ICU* intensive care unit, *IQR* interquartile range

**Fig. 4:**
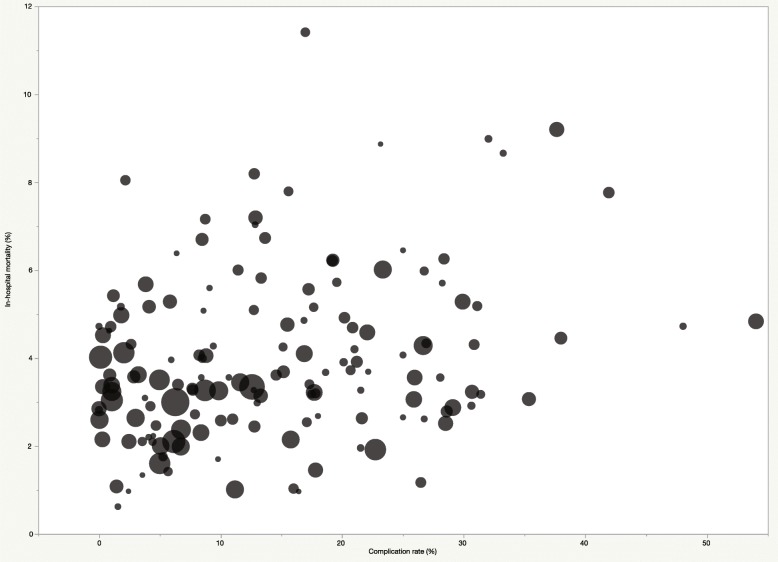
Correlation between complication rate and in-hospital mortality among patients with trauma in all hospitals (*n* = 151). A bubble reflects the hospital, and its size represents the number of patients. The correlation coefficient (*r*^2^) between complication rate and in-hospital mortality was 0.2727 (*P* < 0.01) in all hospitals

**Fig. 5:**
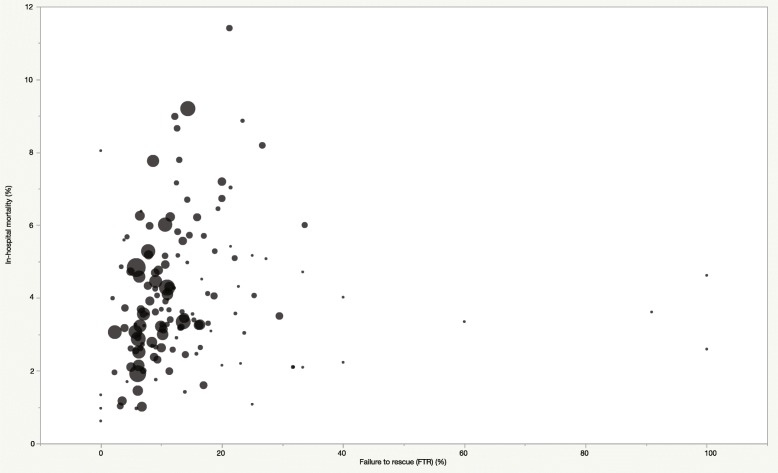
Correlation between failure-to-rescue (FTR) and in-hospital mortality among patients with trauma in all hospitals (*n* = 148). Each hospital was a bubble, whose size reflects the number of patients with any complication. Only three hospitals reported no patients with any complication. The correlation coefficient (*r*^2^) between FTR and in-hospital mortality was 0.0716 (*P* = 0.39) in all hospitals

The correlation coefficient (*r*^2^) between complication rate and in-hospital mortality was 0.2728 (*P* < 0.01) for hospitals contributing at least 20 patients with complications to the cohort complications (*n* = 128) and 0.2727 (*P* < 0.01) in all hospitals (*n* = 151). Figure 4 shows the correlation bubble plot. The correlation coefficient (*r*^2^) between FTR and in-hospital mortality was 0.2766 (*P* < 0.01) for hospitals contributing at least 20 patients with complications to the cohort complications (*n* = 128) and 0.0716 (*P* = 0.39) in all hospitals (*n* = 148). Figure 5 shows the correlation bubble plot.

## Discussion

Our study indicated that complication rates and FTR were associated to in-hospital mortality rates at the center level, as previously reported [7]. Better patient care in high-performing trauma hospitals could be related to a lower risk of complications and rescue from a complication.

In-hospital mortality among trauma patients with complications (FTR) was almost two times more in high-mortality hospitals compared to low-mortality hospitals, similar to a previous study [7]. Our findings are also in line with another study [1], which was higher in high-mortality hospitals compared with low-mortality hospitals. However, previous studies [1, 7] showed discordant results regarding the complication rates. A study showed both lower complication rate and lower FTR related to patient’s better outcomes [7], but another study showed lower FTR related to patient’s better outcomes despite the similarity in complication rates in each hospital [1]; this was consistent with a previous report on elective surgery [3]. Our study supported the former [7]. Successful rescue of patients with complications after trauma would have led to lower mortality rates in high-performance hospitals. To improve the quality of trauma care, it is important not only to survive the trauma but also to avoid complications, and to be rescued from complications as well. Therefore, FTR is a reasonable measure of hospital quality that is strongly related to mortality.

A previous report on elective surgery concluded that complications and mortality are not correlated at the hospital level [3]. They argued that the focus should not be on improved operative techniques to prevent complications but on more efficient rescuing from the complications. In fact, neither our data nor the report by Haas et al. [7] replicates Ghaferi’s results [3]. Trauma complication studies, including our study, recruited not only patients who underwent surgery, but also patients who underwent nonoperative management. Nonoperative management for trauma care has been increasingly mainstream every year. Most of trauma surgeries are also emergency cases. Moreover, complications of trauma are not the same as those with elective surgeries. Therefore, the management of inpatients after trauma such as pneumonia is important, even if they did not undergo surgery.

Treatments and interventions differed with hospital performance. High-mortality hospitals had more emergency procedures but fewer surgeries. Though interventions may be related to the occurrence of a complication, there have been no studies investigating this relationship. Unfortunately, we did not have data on the adequacy of any procedure. There were various complications after trauma in our study, with infections and coagulopathy being the most common, but these complications did not show clinical difference according to hospital performance of trauma care. Indeed, specific complications included in studies have varied over time [14]. Though we captured trivial complications compared to other previous FTR [14], selection of complications was similar to other FTR studies in trauma patients [11]. It is plausible that not only major complications, but also trivial complications, may be related to worse outcomes.

The findings from the current and previous studies [1, 7, 15] add to the list of growing evidence showing that management of complications is central to health outcomes. A retrospective observational study on non-trauma patients showed that low FTR hospitals had significantly more staffing resources than high FTR hospitals [4]. One study showed surgical intensivists benefited trauma patients [16]. Taken together, these findings highlight the importance of closed intensive care unit staffing (nursing, staffing, education, work environment), a higher proportion of board-certified intensivists, and inpatient support in terms of hospitalists, residents including those with teaching status, overnight care, and dedicated rapid response team in trauma practice. Though staffing and management data were not available for extraction in JTDB, these variables may have been related to reduction trauma surgery with a corresponding increase in endovascular treatment and intensive care. Others have reported that sophisticated technology and larger volumes of hospital and surgeons were modifiable hospital factors that improved FTR, although patient’s factors were also related to FTR [14]. A team-based multidisciplinary approach could play an important role in trauma care by reducing judgment errors, delays in diagnosis of trauma, and crucial complications due to errors [17].

### Limitations

This study is not without limitations. First, the complications lacked data on the date of occurrence and the context of each complication was unknown. However, we assessed the timing of the complications based on the type and nature of complications. Some complications like internal diseases might have caused the trauma. Second, complications may have been under-reported leading to underestimation or misclassification. Considering the difference in the correlation coefficient in all hospitals and specifically those that contributed to the study, it is plausible for some hospitals to have misdiagnosed or misregistered complications to JTDB. This was corroborated by the authors of a previous study who reported on the inadequate registration of complications [7]. However, another study found no meaningful differences between a registry and a chart review [18]. Thus, fair and accurate reporting of complications is essential for estimating hospital performance. Third, the impact of small hospitals is not known because we excluded hospitals that contributed little to JTDB. Fourth, we did not extract data related to treatments after complications. Fifth, we did not have the data on the type of care provided in different hospitals. We could not specify the type of care administered (unit, team, hospital characteristics, etc.) and as such could not help addressing the much needed better understanding of what made one hospital really better than another. In addition, we could not show which factors lead to better outcomes. Therefore, it might be difficult to identify what we could change at their own institution to improve outcomes. However, we know the importance of prevention of and rescue from complications.

## Conclusions

Thus, complication rates and FTR are associated with in-hospital mortality rates at the center level, unlike reports of elective surgery. Better patient care in high-performing trauma hospitals could be related to a lower risk of complications and rescue from a complication.

## Data Availability

The datasets analyzed during the current study is available with the corresponding author on reasonable request.

## Abbreviations

FTR: Failure-to-rescue
ISS: Injury severity score
JTDB: Japan Trauma Data Bank
ED: Emergency department
AIS: Abbreviated injury scale
IQRs: Interquartile ranges
